# Use of Antimony in the Treatment of Leishmaniasis: Current Status and Future Directions

**DOI:** 10.4061/2011/571242

**Published:** 2011-06-08

**Authors:** Arun Kumar Haldar, Pradip Sen, Syamal Roy

**Affiliations:** ^1^Division of Infectious Diseases and Immunology, Indian Institute of Chemical Biology, Council of Scientific and Industrial Research, 4 Raja S. C. Mullick Road, Kolkata West Bengal 700032, India; ^2^Division of Cell Biology and Immunology, Institute of Microbial Technology, Council of Scientific and Industrial Research, Chandigarh 160036, India

## Abstract

In the recent past the standard treatment of kala-azar involved the use of pentavalent antimonials Sb(V). Because of progressive rise in treatment failure to Sb(V) was limited its use in the treatment program in the Indian subcontinent. Until now the mechanism of action of Sb(V) is not very clear. Recent studies indicated that both parasite and hosts contribute to the antimony efflux mechanism. Interestingly, antimonials show strong immunostimulatory abilities as evident from the upregulation of transplantation antigens and enhanced T cell stimulating ability of normal antigen presenting cells when treated with Sb(V) *in vitro*. Recently, it has been shown that some of the peroxovanadium compounds have Sb(V)-resistance modifying ability in experimental infection with Sb(V) resistant *Leishmania donovani* isolates in murine model. Thus, vanadium compounds may be used in combination with Sb(V) in the treatment of Sb(V) resistance cases of kala-azar.

## 1. Introduction

Leishmaniasis threatens about 350 million men, women, and children in 88 countries around the world. WHO estimates the worldwide prevalence to be approximately 12 million cases, with annual mortality of about 60,000 (http://www.who.int/vaccine_research/diseases/soa_parasitic/en/index3.html#disease%20burden) and around 1-2 million estimated new cases per year (http://www.who.int/leishmaniasis/en/).

Leishmaniasis is caused by a protozoan parasite of the genus *Leishmania *which multiplies in certain vertebrates that act as reservoirs of the disease. The parasite is transmitted to humans through the bite of sandflies that have previously fed on an infected reservoir. The outcome of the disease, however, depends on the species of *Leishmania *causing the infection and the immune response raised against that infection. The cutaneous form tends to heal spontaneously leaving the scars, which may evolve into diffuse cutaneous leishmaniasis, recidivans leishmaniasis, or mucocutaneous leishmaniasis (MCL) depending on the species of *Leishmania *causing infection. Accordingly, patients suffer from disastrous aesthetic consequences. Whereas cutaneous leishmaniasis (CL) is the most common form of leishmaniasis, visceral leishmaniasis (VL) is the most severe one. In fact, VL can be fatal when left untreated and may cause epidemic outbreaks with a high mortality rate. A varying proportion of visceral cases can also evolve into a cutaneous form known as post-kala-azar dermal leishmaniasis (PKDL), which requires lengthy and costly treatment. Depending on the geographical areas, a specific form of Leishmaniasis may be caused by different *Leishmania* spp. For example, CL and MCL in Central and South America are caused by *L. mexicana* and *L. braziliensis* whereas CL in South and Central Asia and the Middle East is caused by *L. tropica *and *L. major*. Similarly, VL (commonly known “kala-azar”) is caused by *L. donovani* in India, Bangladesh, China, Nepal, and Sudan, by* L. infantum* in North Africa and southern Europe, and by *L. chagasi* in Latin America (http://www.who.int/leishmaniasis/en/). The majority of MCL cases occur in Bolivia, Brazil, and Peru. 90% of CL cases occur in Afghanistan, Brazil, Iran, Peru, Saudi Arabia, and Syria. Under immunosuppressive conditions such as acquired immunodeficiency syndrome (AIDS), dermotropic species of *Leishmania* parasite has also been reported to visceralize to give rise VL. Because human immunodeficiency virus (HIV)-1 is a frequent cause of immunosuppression, an increasing number of cases of HIV-*Leishmania *coinfection are being reported in areas where both infections overlap (geographical distribution of leishmaniasis. Geneva: WHO.Available at: http://www.who.int/emc/diseases/leish/leisgeo.html). In addition, HIV modifies the clinical presentation of all forms of leishmaniasis in the coinfected patients. 

As noted above, some forms of leishmaniasis, for example, VL might be fatal for patients if left untreated. In the absence of an effective vaccine, the control of leishmaniasis is solely dependent on chemotherapy. The organoantimonial compounds have remained as the first line of treatment for all forms of leishmaniasis for more than 60 years. However, until recently, little is known about the chemical structure of these compounds and the methods used in the industry for their preparation [1]. Furthermore, molecular and cellular mechanisms of their action are not well defined. In recent years, a large-scale increase in clinical resistance to pentavalent antimonials has been reported [2, 3]. In India, 65% of previously untreated patients fail to respond promptly or relapse after therapy with antimonials [4].

Second-line drugs include pentamidine and amphotericin B, but severe side effects and high cost limit their use [5]. Miltefosine (hexadecylphosphocholine), originally developed as an anticancer agent, has now been approved as the first oral drug for leishmaniasis. It can be used for both antimony-responding and nonresponding patients [6]. Although it shows good efficacy, but it is very expensive and has a long half-life. Data from phase IV clinical trials in India involving domiciliary treatment with miltefosine along with weekly supervision suggest a doubling in the relapse rate against miltefosine [7]. Beside miltefosine is found to be a potential teratogen in animals. Since there are very few affordable drugs in hand, resistance to first-line drug(s) has a very big impact on the treatment of leishmaniasis. This demands an understanding of the molecular and biochemical mechanisms of clinical resistance, which has become a World Health Organization priority (http://www.who.int/tdr/diseases/leish/strategy.htm).

## 2. Treatment of Leishmaniasis and Antimonials

### 2.1. Historical Perspective of the Disease and Therapy

Historically, the cutaneous form of leishmaniasis is a disease of antiquity and was recognized in the Old World with various names such as oriental sore, Delhi boil, Baghdad sore, and so forth. This is an ancient disease. Descriptions of conspicuous lesions have been found on tablets in the library of King Ashurbanipal from the 7th century BC, some of which are thought to have been derived from earlier texts dating from 1500 to 2500 BC. In addition, in the 10th century Arab physicians have described the oriental sore [8, 9]. Similarly, the visceral form of leishmaniasis in the Old World had been known with various other names like Jessore fever, Kala-dukh, Sarkari Beemari, Dumdum fever, Burdwan fever, Fatal-fever and kala-azar (kala-black; azar- fever). The earliest kala-azar epidemic occurred in 1824 in Jessore district of India (now in Bangladesh), which had initially been confused with malaria and named as Jessore fever [10]. This epidemic killed several thousands of patients because no treatment was known until then. The cutaneous leishmaniasis was used to be treated by local therapy in the endemic areas. However, by the end of 19th century in Tashkent, pure lactic acid was applied to the lesions to cauterize it [11]. Relapses were treated by removal “scraping” of the lesion with a sharp spoon. Other cauterizing agents included copper sulfate, old battery acid, plant extracts and heating of the lesions for 20 hours with water in circulating water baths [11]. The visceral form of the disease was often diagnosed by enlargement of abdomen and was anecdotally treated in India by burning the abdominal skin over the spleen. 

Antimony has been used as therapeutics in several centuries. Some authors have suggested its earliest use in ancient Egypt for cosmetic purposes. However, it has been shown that this statement was based on a misreading of the ancient texts [12]. The importance of antimony in the early medicine is well documented, due to the debate created around their utilization in this period [13]. Paracelsus introduced antimony, as a general panacea in the 16th century (as published in Leipzig in 1604), and it was acclaimed as one of the seven wonders of the world. The modern era of usage of antimony began in 1905 when Plimmer and Thompson showed the activities of sodium and potassium tartrate against trypanosomes; subsequently these were used for the treatment of human trypanosomiasis in Africa. Use of the trivalent antimonial, tarteremetic was first reported for the treatment of CL by Vianna in 1913 [14], the efficacy was confirmed against VL by Di Cristina and Caronia in Sicily [15] and Rogers in India in 1915 [16], but later this drug was found to be highly toxic as well as very unstable in tropical climate [17]. Shortt from India was not impressed with the outcome and wrote that antimony tartrate, is an advance over no treatment at all, rather suboptimal in terms of clinical resistance and relapses [18]. In another report Cole [11] also concluded that tartar emetic was “not much better than no treatment at all.” Tartar emetic was considered as an irritating drug, since it exhibited side effects such as cough, chest pain and great depression. This led to the discovery of pentavalent antimonials. Thereafter, the pentavalent antimony compound urea stibamine synthesized by Brahmachari, emerged as an effective chemotherapeutic agent against Indian kala-azar (KA) in 1920 [19, 20]. This discovery saved millions of lives of poor Indians, for which Professor Brahmchari was nominated for the Nobel Prize in 1929 (Nobel Prize official website.) [10]. The development of the less toxic pentavalent antimonials by Brahmachari, Schmidt, Kikuth, and others led to the synthesis of antimony gluconate (Solustibosan) in 1937 [21] and sodium stibogluconate (Pentostam) in 1945 [22]. Now a days the most commonly used organic compounds of antimony (Sb) are sodium antimony gluconate (SAG; manufactured by Albert-David, Kolkata, India) and meglumine antimoniate (manufactured by Rhone-Poulence, Paris).

### 2.2. Structure

Structures of two complexes of Sb(V) with *N*-methyl-D-glucamine (meglumine antimoniate or Glucantime) or sodium gluconate (sodium stibogluconate or Pentostam) remained unknown for decades due to their amorphous state. Recently, NMR and mass spectrometric approaches have allowed significant progress in this arena [1].

**Figure 1:**
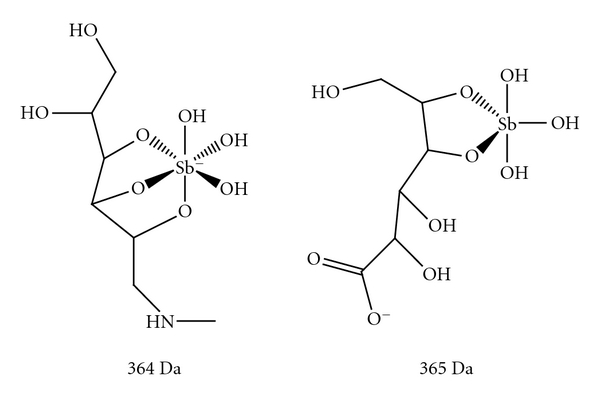
Proposed structural formula for 364 Da and 365 Da ions identified by ESI (−)-MS in aqueous solutions of meglumine antimoniate and stibogluconate, respectively. Adapted from [25].

Fast-atom bombardment mass spectrometric (FAB-MS) data of the commercially available meglumine antimoniate suggests that two molecules of meglumine (NMG) coordinate with a single Sb atom in a symmetrical geometry [23]. On the other hand, positive ion electrospray ionization mass spectrometric (ESI(+)-MS) analyses indicate the existence of a mixture of polymeric structures with the general formula (NMG-Sb)*n*-NMG [24]. Further analyses of meglumine antimoniate by ESI-MS, in both the positive and negative modes, show negatively charged 1 : 1 (*m/z *364) and 2 : 2 (*m/z *765) Sb(V)-meglumine complexes and support the predominance of zwitterionic species in solution (Figure 1) [25]. ESI-MS measurements of sodium stibogluconate also showed that it consists of a mixture of oligomeric structures [25] that confirmed the earlier results obtained by molecular sieve chromatography [26], and is consistent with the general formula for meglumine antimoniate ((GLU-Sb)*n*-GLU and (GLU-Sb)*n *(GLU: gluconate). Osmolarity measurement suggested the predominance of 1 : 1 Sb-NMG and Sb-SSG complexes in diluted samples [25]. This interpretation was further in agreement with the HPLC-inductively coupled plasma-MS and ESI-MS analyses [27].

### 2.3. Entry of Drug

Pentavalent arsenate (As(V)), a metal related to (Sb(V)), is known to enter via a phosphate transporter [28]. Antimony transport was first studied in both stages of *Leishmania mexicana* and *Leishmania donovani *parasites using (^125^Sb) Pentostam (Sb(V)) [29]. More recently, mass spectroscopic approaches reveal the accumulation of two forms of antimony (Sb(V) and Sb(III)) in both stages of the parasite. However, accumulation of Sb(V) has been found to be higher in axenic amastigotes than in promastigotes in a number of species [30, 31]. Because gluconate competitively inhibits uptake of Sb(V) in axenic amastigotes, Sb(V) is speculated to enter into the parasites via a protein that recognizes a sugar moiety-like structure shared with gluconate [32]. Interestingly, neither As(V) nor phosphate can compete with the uptake of Pentostam in this scenario. This ruled out the possibility that Sb(V) uses an As(V) transporter. However, the accumulation of Sb(III) is competitively inhibited by the related metal As(III) [32], suggesting that Sb(III) and As(III) enter the cell via the same route. 

### 2.4. Mechanisms of Action

Pentavalent antimonials are in use against leishmaniases for more than six decades. However, their molecular and cellular mechanisms of action are not yet well understood. It is not even clear whether the final active form is Sb(V) or Sb(III). Three main models could be proposed regarding the mechanism of action of pentavalent antimonials. 

#### 2.4.1. Prodrug Model

According to this model, pentavalent antimony (Sb(V)) behaves as a prodrug, which undergoes biological reduction to much more active/toxic trivalent form of antimony (Sb(III)) that exhibits antileishmanial activity. However, the site of (amastigote or macrophage) and mechanism of reduction (enzymatic or nonenzymatic) remain controversial. Furthermore, the ability of Leishmania parasites to reduce Sb(V) to Sb(III) is stage-specific. For instance, amastigotes but not promastogotes can reduce Sb(V) to Sb(III). This explains why amastigotes are more susceptible to Sb(V) but promastigotes are not [33–37]. Other studies have suggested that reduction of Sb(V) to Sb(III) may also take place within macrophages, but level of reduction of Sb(V) to Sb(III) in macrophage can not be that significant since Sb(III) even at a dose of ~25 *μ*g/mL can kill 50% of the THP1 macrophages [38, 39]. Thus, conversion of Sb(V) to Sb(III) may occur at both sites, that is, macrophage and parasite, and the parasite plays a major role in the generation of higher, lethal concentrations of Sb(III) within the parasite. It has been shown that, a proportion of Sb(V) may be converted to Sb(III) in human [36, 40] and animals models [41, 42]. 

The reduction of Sb(V) to Sb(III) requires an active participation of thiol compounds of both mammalian host and parasite origin [43–45]. Mammalian thiols, which play important role in this process, include glutathione (GSH), cysteine (Cys) and cysteinyl-glycine (Cys-Gly). The first one is the main thiol present in the cytosol, while the second and the third are the predominant thiols within lysosomes of mammalian cells [46, 47]. The parasite-specific thiol compund, trypanothione (T(SH)2), is a complex consisting of glutathione and spermidine, has been shown to be involved in reduction of Sb(V) to Sb(III) [48]. Compared to GSH, however, the initial rate of reduction of Sb(V) is much higher in the presence of Cys-Gly, Cys, and T(SH)2 [43]. Generally, acidic pH and slightly elevated temperature favor reduction of Sb(V) to Sb(III).* In vivo* this process is mediated by T(SH)2 within *Leishmania *parasites and Cys or Cys-Gly within the acidic compartments of mammalian cells. But the stoichiometry of GSH and Sb(V) required for the reduction of antimony is equal to or more than 5 : 1. As the rate of reduction is very low, the physiological relevance of this conversion is still open to question [49]. 

 Interestingly, promastigotes contain higher intracellular concentrations of T(SH)2 and GSH than amastigotes [50, 51], and both stages maintain an intracellular pH value close to neutral [52]. Therefore, nonenzymic reduction of Sb(V) to Sb(III) fails to account for the insensitivity of promastigotes to Sb(V). On the other hand, recent studies have suggested the participation of an parasite-specific enzyme, thiol-dependent reductase (TDR1), in the process of reduction of Sb(V) to Sb(III) [53]. The enzyme TDR1 is a tetramer protein, containing domains of the omega class of the glutathione S transferases (GSTs), and using GSH as the reductant. Although TDR1 has been found to be highly abundant in the amastigote stage of the parasite, the enzyme activity and antimony sensitivity in Leishmania amastigotes could not be directly correlated.

An arsenate reductase homologue in *Leishmania* parasite (LmACR2) has also been shown to catalyse the reduction of Sb(V) in *L. major* in presence of GSH. LmACR2 requires glutaredoxin as cofactor for its enzyme activity and is inhibited by As(III), Sb(III) and phenylarsine oxide [54]. In contrast to TDR1, LmACR2 is a monomer. Transfection of LmACR2 in *Leishmania infantum* promastigotes augments pentostam sensitivity in intracellular amastigotes, confirming its physiological significance. It is also possible that more than one mechanism is responsible for the reduction of Sb(V) to Sb(III).

**Mechanism of Killing by Reduced Sb(III)**

Trypanothione reductase (TR) and zinc-finger protein are the potential molecular targets of Sb(III). Such interaction is consistent with the modality of Cys binding of thiophilic metals such as As(III), Sb(III), and Bi(III). Metal-bound Cys systems are fully deprotonated thiolate anions, the nucleophilicity of which is greatly attenuated upon formation of metal complexes with high thermodynamic stability.

**Action on Trypanothione/TR System**

Trypanothione/TR system keeps T(SH)2 in the reduced state and thereby maintains oxidoreductive balance in Leishmania parasite. This protects the parasites from oxidative damage and toxic heavy metals, and delivers the reducing equivalents for DNA synthesis [55]. Although TR shares structural and mechanistic similarity with glutathione reductase (GR), differences in the disulfide binding site between TR and GR account for selective inhibition. Trivalent antimonials interfere with T(SH)2 metabolism by inhibiting TR and inducing rapid efflux of intracellular T(SH)2 and GSH into intact *Leishmania *cells [51, 56]. Recently, it has been shown that Sb(III) can bind to a CCHC zinc-finger peptide model and promote the ejection of Zn(II) [57]. The zinc-finger domain is characterized by the coordination of a zinc atom by several amino acid residues, which are usually cysteines and histidines. These structural elements are associated with protein-nucleic acid and protein-protein interactions [58]. The CCHC motif bearing Zn finger proteins binds to the hexanucleotide repeat sequence found in the intervening region of the GP63 (most abundant surface glycoprotein) gene cluster of Trypanosomatids. Zn finger proteins are likely to be involved in DNA replication, structure and repair [59]. Treatment of *Leishmania *amastigotes with Sb(III) has been found to induce apoptosis via induction of the oxidative-stress and increase in intracellular (Ca^2+^) [60, 61].

#### 2.4.2. Intrinsic Antileishmanial Activity Model

According to this model, Sb(V) has intrinsic antileishmanial activity. Initial studies suggested that sodium stibogluconate [Sb(V)] inhibits macromolecular biosynthesis in amastigotes [62], possibly via perturbation of energy metabolism due to inhibition of glycolysis and fatty acid betaoxidation [63]. However, the specific targets in these pathways have not been identified. Sodium stibogluconate, but not Sb(III), specifically inhibits type I DNA topoisomerase, thus inhibiting of unwinding and cleavage. Sb(III) mediated inhibition seems to be specific for *Leishmania donovani *topoisomerase, since Sb(III) fails to inhibit calf-thymus topoisomerase I and *Escherichia coli *DNA gyrase [64, 65]. Interestingly, *in vivo *sensitivity and resistance of *Leishmania *towards antimonial drugs have been shown to correlate with the effect of such a complex [66].

 Demicheli and coworkers have reported the formation of a complex between adenine nucleosides and Sb(V) [67]. Formation of Sb(V)-ribonucleoside complexes, both in the ratio of 1 : 1 and 1 : 2 was evidenced [68, 69]. The large changes for H2′ NMR resonance suggested that –OH groups in the ribose are the binding sites for Sb(V) probably via ring chelation at C2′ and C3′. Complex formation between ribonucleosides and Sb(V) was found to be faster at acidic pH, indicating that it is kinetically favored in acidic biological compartments. The rate of dissociation is slow in aqueous solutions at neutral pH. Moreover, the stability constant determined for 1 : 1 Sb(V)-GMP complex is consistent with the formation of such a complex in the vertebrate host following treatment with pentavalent antimonial drugs, especially if the high accumulation and prolonged retention of antimony in macrophages is considered [70, 71]. Regarding the possible pharmacological role of Sb(V)-ribonucleoside complexes, two hypotheses may be raised: (a) formation of Sb(V)-adenine nucleotide complex might act as an inhibitor of the *Leishmania *purine transporters, or (b) these complexes might penetrate inside the parasite, encountering a neutral pH-environment where dissociation gets retarded and the complex as such behaves like the purine analog (as allopurinol), thus interfere with the purine salvage pathway [72, 73].

#### 2.4.3. Host Immune Activation Model

According to this model antimonials clear intracellular Leishmania parasites via activation of host immune system. Action of sodium antimony gluconate (SAG) is multifaceted. SAG can activate both innate as well as adaptive immune compartments, thereby inducing effective antileishmanial immune response. This not only ameliorates existing infection but also protect from relapse.

**Effect on Innate Immunity**

Croft and Yardley 2002 [74] mentioned a moderate role for antimonial action in the paradigm “the reticuloendothelial system (i.e., its stimulation by drugs, etc.) is of importance for the cure.” Murrayand Nathan [75] demonstrated that MΦ activation had a significant effect on intracellular parasite killing. Treatment with SAG has been reported to induce ROS generation in peripheral blood cells of *L. infantum *infected mice on stimulation with phorbol ester (PMA) or zymosan [76], and to induce NO in canine leishmaniasis [77]. Recently, it has been reported by us [78] that SAG alone can induce both ROS and NO production in murine MΦ and promote two waves of  killing of *L. donovani* amastigotes. The first phase of killing (i.e., at early time point, around 6 h post treatment) is due to induction of ROS and the second wave of killing (i.e., at a later time point, 24 h and 48 h) is mediated by NO generation. Both ROS and NO are known to be involved in parasite killing in the early stage of leishmanial infection in mice, whereas NO alone is involved in the late phase [79, 80]. The role of NO in final elimination of leishmanial parasites is further strengthened by the studies which demonstrated that treatment of *L. major* infected mice with L-NMMA drastically increases the lesion size and *L. major* is visceralized in a late phase of experimental infection in iNOS knockout mice [81].

**SAG Mediating Activation of Signaling Pathways**

We further deciphered the signaling mechanisms responsible for SAG-induced ROS and NO production and consequent killing of intracellular leishmania parasites within infected MΦ. SAG-induced ROS generation in MΦ requires phosphorylation of ERK via the PI3K-PKC-Ras/Raf pathway. On the other hand, activation of the PI3K/Akt pathway and downstream p38MAPK is essential for induction of NO production and subsequent parasite killing in* L. donovani*-infected MΦ following SAG treatment. It was further shown that p38MAPK mediated generation of NO by SAG treatment is an indirect mechanism. Actually p38MAPK induces TNF*α* production, which in turn induces iNOS2 expression and subsequent NO generation since SAG-mediated NO generation and parasite killing could be abrogated by treatment with antiTNF*α* neutralizing antibody [78].

Leishmania infection has been reported to increase PTPase activity mainly that of SHP1 type [82–84], which might contribute to dysregulation of PTK dependent signaling events and MΦ deactivation. SAG inhibits SHP1 and SHP2 classes of PTPases but not MKP1 type [85] by the gluconic acid backbone bound in various specific stoichiometric ratios inhibit purified SHP1 with specific efficacies. SHP1 might directly dephosphorylate ERKs [86] mechanisms by which *Leishmania* parasites can escape and regulate activation of other important signaling molecules like PI3K. Thus, inhibition of SHP1 by SAG might indirectly favor tyrosine phosphorylation of PI3K and thereby help in activating both PI3K-PKC-Ras/Raf-ERK1/2 pathway for ROS generation and the PI3K-Akt-p38 MAPK pathway for NO generation. In addition, SAG upregulates IFN-*γ* receptors both in uninfected and *L. donovani* infected THP1 cells, as well as in monocytes derived from kala-azar patients treated with SAG [87]. Thus it is quite possible that SAG influences the host's antileishmanial defense by altering IFN-gamma responsiveness. Indeed, SAG fails to act in IFN-*γ* knockout mice [88]. We have also observed that SAG and IFN-*γ* synergize to produce high levels of NO in MΦs. A combination of SAG and IFN-*γ* is also known to be therapeutically much more effective than SAG alone in the treatment of visceral leishmaniasis [89]. We have further observed that SAG triggers production of IL12 in both uninfected as well as infected MΦ. IL12 is known to induce Th cells to produce IFN-*γ*, which in turn activates MΦs to produce TNF-alpha and, subsequently, NO.

### 2.5. Effect of Antimony on Cell Mediated Immunity 

#### 2.5.1. Action on T Cell

Studies of murine VL infections (BALB/c-*L. donovani*) have established that an intact T-cell population, more specifically Th1, is required for Sb(V) to produce a curative antileishmanial effect [90, 91]. The drug itself is leishmanicidal *in vitro *and *in vivo*, however complete cure, *in vivo*, is not achieved without Th1 input. Patients coinfected with VL-HIV respond poorly to antimony treatment [92]. After an initial response, these patients frequently relapse and require alternative treatment [93]. Dermotropic infections in man usually self-cure. This can take from 3 months to 3 years depending on the species of *Leishmania *involved. In such cases antimonial treatment augments the host's immune response to rapidly resolve the infection. Exceptional cases include DCL where, in the absence of a cell mediated response, antimonials prove to be ineffective [94]. Several studies have shown that endogenous IL-2 [95], IL-4 [96, 97] and IL-12 [98] influence the effectiveness of chemotherapy with pentavalent antimony. These findings indicate the requirement of somewhat functional T cell compartment for SAG action.

**Figure 2:**
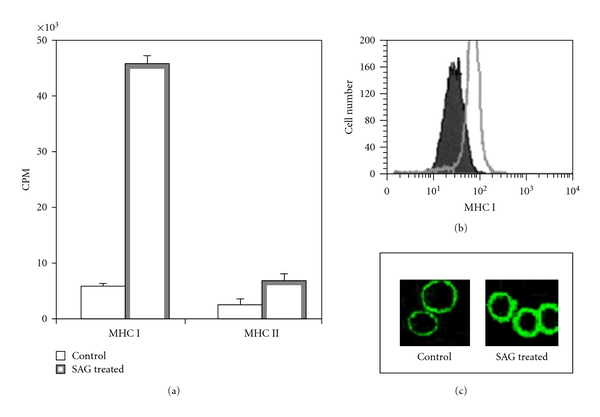
SAG increases MHC class I mediated antigen presentation and upregulates expression of MHC class I. MΦs isolated from BALB/c and C57BL/6 mice, cultured in presence or absence of SAG for 24 h. (a) To study the antigen presenting function, peritoneal MΦs from BALB/c and C57BL/6 mice either kept untreated or treated with SAG for 24 h, were used as antigen presenting cells to drive the T-cell hybridoma in presence of appropriate peptide and IL-2 secretion was tested on IL-2-dependent cell line (HT-2). The growth of HT-2 was studied using ^3^H-Thymidine incorporation. The studies showed that class I but not class II restricted presentation was significantly (*P* < .001) enhanced upon SAG treatment both in normal and infected MΦ. (b) To study the expression of MHC I molecules, untreated (filled histogram) and SAG-treated (open histogram) MΦs from BALB/c mice were stained with FITC labeled anti-D^d^ (BD Pharmingen) according to manufacturer's instruction and either analyzed on flow cytometer or examined under a confocal laser scanning microscope. The studies showed that class I expression was significantly (*P* < .001) enhanced upon SAG treatment. Antigen presentation assay was performed at least thrice and the results are presented as mean ± SD. For flow cytometry and confocal microscopy, representative data of 3 similar experiments is presented here.

Our study indicates that effect of SAG on T cell compartment is corollary to its action on antigen presenting cells like MΦ. We observed that SAG treatment enhances expression of specifically MHC I molecule on the MΦ surface and enhanced class I mediated antigen presentation, but not the presentation mediated by MHC class II (Figure 2). This may be a mechanism by which SAG can enhance antileishmanial cytotoxic T lymphocyte (CTL) response. There is a report that CTLs can kill intracellular parasites [99].

 Interestingly stimulation of spleen cells, derived from either *Leishmania* infected or uninfected mice, induced IFN-*γ* generation (Mookerjee Basu, unpublished data). Carter et al. showed that SAG treatment of infected mice imparted resistance to reinfection while SAG treatment prior to infection imparted partial resistance to *Leishmania* infection. 

**Figure 3:**
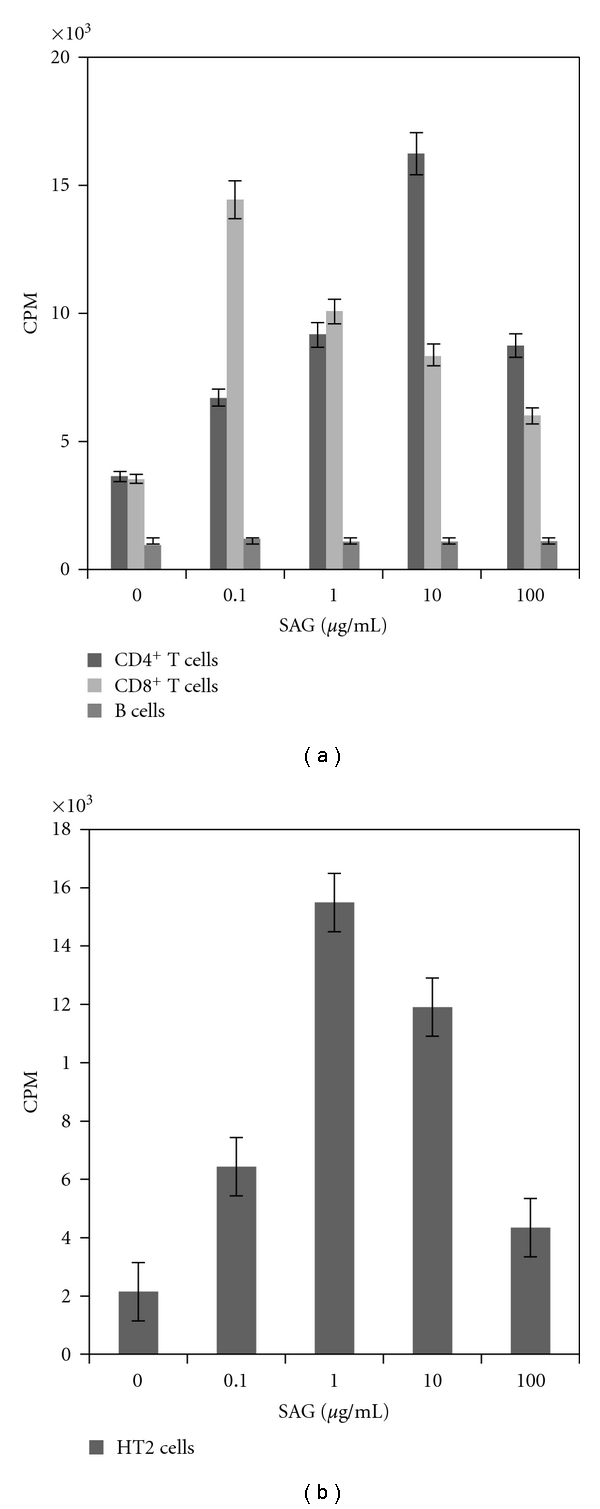
SAG directly stimulates proliferation of T cells. 10^5^ lymphocytes, from normal BALB/c mice (a) and 5 × 10^4^ IL-2-dependent CD8^+^ cytotoxic T cell line (CTLL-2) were plated in each well and were kept either untreated or treated *in vitro* with various concentrations of SAG. Proliferation of each type of cells was monitored by ^3^H thymidine incorporation. Each experiment was performed at least thrice and results are presented as mean ± SD.

SAG-induces proliferation of T-cells but not of B cells (Figure 3) even in absence of antigen presenting cells (Mookerjee-Basu, unpublished observation). Interestingly SAG-mediated proliferation of T cells does not require IL-2 (Figure 3). 

Thus on the one hand SAG could activate T cell compartment (in both MHC-independent and -dependent manner), and on the other could directly activate MΦs to induce generation of microbicidal effector molecules (ROS and NO) which in concert help to potentiate both innate and cellular arms of immune system to eliminate LD parasites.

## 3. Resistance to Antimonials

### 3.1. Clinical Resistance

Pentavalent antimonial drugs were used worldwide for the treatment of VL and CL for over six decades with little evidence of resistance. There is a regional variation in response to antileishmanial drugs and thus recommendations for treatment of VL vary in different regions. Although the selection of resistant *Leishmania *has long been a part of laboratory studies, it is only in the past 15 years that acquired resistance has become a clinical threat. Pentavalent antimonials remain the treatment of choice in Africa, South America, Bangladesh, Nepal, and India (except North Bihar) at the dose of 20 mg/kg/day parenterally for 28–30 days. In the Mediterranean basin liposomal amphotericin B (L-AmB) is the treatment of choice for immunocompetent patients [100]. The drug of choice for the treatment of HIV/VL coinfection is an extended course of L-AmB [101]. However, the region endemic for VL in North Bihar, India, has the unique distinction of being the only region in the world where widespread primary failure to Sb(V) has been reported [102]. Even in this geographical region a variation in Sb(V) sensitivity occurs with significant drug resistance at the epicenter of the epidemic and a high level of sensitivity only 200 miles away [103]. This resistance is so far unique to *L. donovani*; all isolates from a large number of refractory as well as responding patients in India were identified as this species [4].

### 3.2. History of Antimony Resistance

Until the late 1970s, a small daily dose (10 mg/kg; 600 mg maximum of Sb(V)) for short duration (6 to 10 day) was considered adequate. In an earlier resurgence of Indian VL, which assumed epidemic proportions by 1977, an estimated 250,000 patients were affected in Bihar, when unconfirmed reports suggested a 30% treatment failure with this regimen from the four districts most severely affected, viz Muzaffarpur, Samastipur, Vaishali, and Sitamarhi [104]. Following this, an expert committee revised recommendations to use Sb(V) in two 10-day courses with an interval of 10 days and a significant improvement in cure rates (99%) was observed [105]. However, only a few years later, another study noted 86% cure rates with 20 days of continuous treatment with this regimen [106]. In 1984, a WHO expert committee recommended that Sb(V) should be used in doses of 20 mg/kg/day up to a maximum of 850 mg for 20 days, with a repeat of the same regimen for 20 days in cases of treatment failure. Four years later, Thakur et al. evaluated the WHO recommendations and reported that 20 days of treatment with 20 mg/kg/day (maximum 850 mg) cured only 81% of patients, although with an extension of the treatment for 40 days 97% of patients could be cured (Table 1) [107].

Three years later, the same group noted a further decline in cure rate to 71% after 20 days of treatment, and recommended extended duration of treatment in nonresponders. Mishra et al. [5] found that extending the therapy, to 30 days could cure only 64% of patients in a hyperendemic district of Bihar, while 100 percent resistance cases of kala-azar was observed in two villages of Darbhanga and Sitamarhi districts (182 and 59 cases, resp.). From these findings it became clear that Sb(V) refractoriness was increasing although the reports came from studies that were not strictly controlled. In two following studies carried out under strictly supervised treatment schedules it was observed that only about one-third of all VL patients could be cured with the currently prevailing regimen. The incidence of primary unresponsiveness was 52%, whereas 8% of patients relapsed. During the same period, the treatment failed with only 2% of patients from the neighboring state of (Eastern) Uttar Pradesh [108]. These studies confirmed that a high level of Sb(V) unresponsiveness exists in Bihar, though the drug continues to be effective in surrounding areas. There are reports of antimony resistance spreading to the Terai regions of Nepal, especially from the district adjoining hyperendemic areas of Bihar, where up to 30% of patients seem to be unresponsive, though in eastern Nepal a 90% cure rate has been reported [109].

### 3.3. Reason of Antimony Treatment Failure

The reason for the emergence of resistance is widespread misuse of the drug. Sb(V) is freely available in India. Both qualified medical practitioners and unqualified quacks used the drug and this unrestricted availability of the drug led to rampant misuse. Most patients (73%) first consult unqualified medical practitioners, who might not use the drug appropriately [110]. It has been a common practice to start with a small dose and gradually increase the dose over a week. Drug-free intervals are given with the belief that they will prevent renal toxicity. On many occasions the daily dose of drug is split into two injections, to be given twice daily. These practices presumably expose the parasites to drug pressure, leading to progressive tolerance of the parasite to Sb(V). It has been observed that only a minority of patients (26%) were treated according to prescribed guidelines: irregular use and incomplete treatments were a common occurrence. These facts point to the mishandling of antileishmanial drugs in Bihar as a significant contributor to the development of drug resistance [103].

The growing resistance to Sb(V) in India while it still remained sensitive all over the world could be due to the fact that leishmaniasis usually has zoonotic transmission except in the Indian subcontinent and East Africa where the transmission is largely anthroponotic. In an anthroponotic cycle, once Sb(V) resistance gets established, it spreads exponentially and organisms sensitive to the drug get eliminated quickly, whereas the drug-resistant parasites continue to circulate in the community [111]. 

In CL the response is not as predictable, because there is considerable variation in sensitivity to Sb(V) among primary isolates from untreated patients with cutaneous leishmaniasis, which correlates with patients' response to treatment [112]. Except Bihar, primary resistance is quite uncommon, but resistance develops in patients with VL, CL, and MCL who have relapsed. Chances of response to further courses of antimonials diminish once there is a relapse after the initial Sb(V) treatment [113]. In *L. infantum *isolates taken from VL patients in France, drug-sensitive strains (ED_50_  <  40 *μ*g/mL) were isolated from patients who responded quickly to meglumine treatment, whereas all the strains which were resistant under *in vitro* conditions (ED_50_  >  70 *μ*g/mL) corresponded to clinical failures. *In vitro* sensitivity of strains decreased progressively in relapsing patients treated with meglumine [2].

### 3.4. Cellular and Molecular Mechanism of Antimony Resistance

It is evident from the above discussion that the response towards antimony treatments depends on several factors some are parasite related and some are host dependent.

#### 3.4.1. Resistance at the Level of Parasite

**Species Variation**

Variation in clinical response to the pentavalent antimonials sodium stibogluconate, and meglumine antimonate (Glucantime) in VL, CL, and MCL has been a persistent problem in the treatment of leishmaniasis over the past 50 years. One explanation for this phenomenon is the intrinsic difference in species sensitivity to these drugs. In studies using the amastigote-macrophage model, *L. donovani *and *L. brasiliensis *were found to be three- to fivefold more sensitive to sodium stibogluconate than *L. major*, *L. tropica*, and *L. mexicana *[114]. This was also shown in earlier studies by Berman et al. using another amastigote macrophage model, which also demonstrated a wide variation in the sensitivity of isolates from cutaneous leishmaniasis cases to pentavalent antimonials [112]. In one controlled clinical trial in Guatemala that compared the cure rate to antimonials of CL caused by different species [115], sodium stibogluconate produced a significantly higher cure rate in patients with *L. braziliensis *(96%) lesions than those with *L. mexicana *lesions (57%).

Role of parasites in antimony treatment failure was established using *in vitro* amastigote-macrophage assay using *L. donovani* isolates from responders and nonresponders. Isolates from patients who did respond to sodium stibogluconate treatment were found be threefold more sensitive, with 50% effective doses (ED_50_) around 2.5 *μ*g Sb/mL compared to isolates from patients who did not respond (ED_50_ around 7.5 *μ*g Sb/mL) [3]. The significant differences in amastigote sensitivity supported the concept of acquired antimony resistance in Bihar. 

Other reports on VL isolates from Sudan have also shown that the clinical response to sodium stibogluconate was reflected in isolates in the amastigote-macrophage model (but not in promastigotes) [116]. Other observations support the notion that Sb resistance can be acquired. Of *L. infantum *isolates taken from immunodeficient and immunocompetent VL patients in France both before and after meglumine antimoniate treatment, those from 13 of 14 patients post-treatment had decreased sensitivity in an amastigote-macrophage assay [2]. A similar decreased sensitivity was observed in *L. infantum *isolates taken from dogs before and after meglumine antimoniate treatment [117]. In the laboratory, antimonial resistant* L. donovani* is easily generated in culture, most recently in axenic amastigotes of *L. donovani *and *L. infantum*, but *in vitro* unresponsiveness does not necessarily translate to clinical resistance [118]. Reduction of drug concentration within the parasite, either by decreasing drug uptake or by increasing efflux/sequestration of the drug, constitutes the primary mechanism of antimonial resistance. Other potential resistance mechanisms include inhibition of drug reduction, inactivation of active drug, and gene amplification [119–124].

**Role of Thiol-Metabolism**

Thiol metabolism has a central role in the maintenance of an intracellular reducing environment so that the cell can defend itself against the damage caused by oxidative stress inside the macrophage, oxidants, certain heavy metals and, possibly, xenobiotics [125]. As antimony causes oxidative stress [60, 126], a reducing environment within the cell and the presence of thiols become important for antimony resistance. Arsenite- or antimony-resistant laboratory mutants of all Leishmania species exhibit significantly increased levels of intracellular thiols, namely cysteine, GSH, and trypanothione (TSH), suggesting a role for thiols in resistance [127–129]. The synthesis of two precursors GSH and spermidine determines the level of TSH. The *γ*-GCS gene encoding *γ*-glutamylcysteine synthetase, which catalyses the rate-limiting step in GSH biosynthesis, has been found to be amplified in arsenite-resistant *L. tarentolae* [130], while the gene ODC which encodes ornithine decarboxylase, an enzyme involved in the regulation of spermidine biosynthesis, is also overexpressed [131, 132]. This suggests that a lowering of intracellular thiol concentration may result in the attenuation of the resistant phenotype. This proposed hypothesis is confirmed by inhibition studies. The inhibition of the *γ*-GCS and ODC genes by their specific inhibitors, L-buthionine-(SR)-sulphoximine (BSO) and DL-a-difluoromethylornithine (DFMO), respectively, results in the reversal of arsenite or antimony resistance in laboratory mutants [130, 133]. Although the combination of BSO and DFMO sensitizes the resistant cells, the residual level of resistance is still higher than that in wild-type cells, suggesting that GSH or TSH alone is not sufficient to confer metal resistance. Overexpression of either ODC or *γ*-GCS in *L. tarentolae* wild-type cells results in increased thiol levels, almost equivalent to those of resistant mutants, but the transfectants do not exhibit arsenite resistance [130]. While cotransfection of ODC or *γ*-GCS with MRPA in wild-type cells results in arsenite resistance [129, 132], this acquired resistance in transfectants is also reversed by the thiol depletor BSO [134]. This therefore establishes that MRPA and increased TSH concentrations act synergistically, and that TSH availability is the limiting factor in both the transport of drug conjugates and resistance to arsenite and/or antimony [135]. The tryparedoxin peroxidase family considered to be principally responsible for detoxification of peroxides [136]. The decameric type I tryparedoxin peroxidase (TryP) [137, 138], is a 2-Cys peroxidase, obtaining its reducing equivalents from T(SH)2 via the dithiol protein tryparedoxin (TryX). Studies have associated overexpression of TryP with resistance to both arsenite [139], and antimony [140] in laboratory generated *Leishmania* resistant lines and in-field isolates [141] implying that enhanced antioxidant defences, through overexpression of TryP, may well be a key feature of antimonials resistance. In *Leishmania tropica* and *Leishmania mexicana* cell lines, an increase in TSH is not associated with either the amplification of *γ*-GCS or overexpression of ODC [128]. Interestingly, resistance to Sb(V) in *L. donovani* clinical isolates (India) is also reversed in animal models by treatment with BSO [142, 143]. It is also noteworthy that the expression of *γ*-GCS in these resistant isolates is also increased significantly. Interestingly, in another study on *L. donovani* isolates from Nepal, expression of *γ*-GCS and ODC was significantly decreased in resistant isolates [121]. Therefore, there is a need to study the level of thiols in clinical isolates and determine their role in natural antimony resistance. It was also shown that antimony-resistant isolates downregulate the expression of *γ*-GCS of macrophages [144], probably by downregulating host NF*κ*B, which is known to regulate *γ*-GCS expression [145]. This would result in the reduction of intramacrophage GSH levels and promote an intracellular oxidative environment, thereby minimizing the intramacrophage reduction of Sb(V) to its toxic form Sb(III) [39]. These results clearly indicate that SAG resistance in *L. donovani* is associated with manipulation of both host and parasite thiol levels. Spontaneous formation of Sb(III), complexed with GSH or TSH or both, has already been demonstrated by proton nuclear magnetic resonance spectroscopy [45, 146] and by MS [127]. Since GST is elevated in mammalian cells selected for resistance to arsenite [147], it has been proposed that GST mediates the formation of metalloid thiol pump substrates in Leishmania species also. However, in *Leishmania*, GST is not detectable; rather, a related trypanothione S-transferase activity is observed [148]. Thus, the thiols have a dual role in antimony resistance, that is, sensitization of the parasite by the reduction of pentavalent to trivalent antimony, and promotion resistance by forming conjugates with trivalent antimony for efflux and/or sequestration.

**Efflux of the Drug**

The efflux of a drug or its active derivative is a very common mechanism of drug resistance in bacteria, yeasts and fungi, and various pathogenic protozoa, for example, *Plasmodium falciparum, Entamoeba histolytica, Giardia lamblia, Trypanosoma cruzi, and Trichomonas vaginalis.* This may be the case in Leishmania too. Two types of ABC transporters are known to be responsible for multidrug resistance (MDR) in cancer cells: P-glycoprotein (P-gp) and multidrug resistance-related protein (MRP). P-gp is encoded by the mdr1 gene, which confers resistance to many hydrophobic drugs (MDR), and is characterized by reversion with verapamil and cyclosporine A. In Leishmania, MRP also confers MDR, although this cannot be reversed by conventional MDR modulators; the protein responsible is known as MRP1.

In Leishmania, both classes of ABC transporters have also been reported to be amplified in various species in response to different drugs under laboratory conditions [149].

Analysis of the complete Leishmania genome (http://www.genedb.org/) has revealed eight putative protein homologues belonging to the MRP1 family, known to be involved in thiol-associated efflux and metal resistance in mammalian cells [150]. Two of them appear to be involved in antimony resistance in the parasite. The first one is PGPA (renamed as MRPA). However, Leishmania MRPA is functionally distinct from mammalian MRP, as resistance is not conferred to pentavalent antimonials, zinc and cadmium, or the typical multidrug-resistant P-gp substrates vinblastine and puromycin [151]. The gene has been found to be amplified in a number of laboratory mutants of Leishmania species selected for resistance to Sb(III), Sb(V), and As(III) [152]. Its role in antimony resistance has been confirmed by transfection studies [128]. However, this transporter is not responsible for the drug efflux across the plasma membrane. Rather, it confers resistance by sequestration of metal-thiol conjugates, a mode of metal detoxification in yeast cells [28]. MRPA is localized in membrane vesicles that are close to the flagellar pocket, the site of endo- and exocytosis in the parasite [153]. Overexpression of MRPA has been reported to decrease influx of antimony rather than increase efflux [33], and this may be due to a dominant negative effect through interaction with other membrane proteins. Thus, MRPA is an intracellular rather than an efflux transporter, and may play a major role in antimony resistance [154]. Recently, it has been shown by DNA microarray assay that MRPA is overexpressed in the axenic amastigote stage of Sb(III)-resistant *L. infantum* [134]. Transfection of MRPA confers Sb(III) resistance in promastigotes and Sb(V) resistance in the intracellular stage of *L. infantum*. However, MRPA has not been found to be upregulated in a comparative transcriptomic study of antimony-resistant *L. donovani* field isolates [121].

Further, no reports are available regarding the amplification of ABC transporter gene(s) in-field isolates. Thus, it is still of great interest to determine whether or not drug-resistant field isolates adopt the same strategies to resist antimony as the laboratory mutants. A second ABC transporter protein (PRP1), involved in antimony resistance, has been isolated by functional cloning selecting for pentamidine resistance [155]. This protein has been shown to confer cross-resistance to antimony. The localization of this protein and the mechanism by which it confers resistance remain to be determined. Another transporter that confers antimony resistance by an active extrusion system independent of MRPA is also present in *L. tarentolae* laboratory mutants [156]. Using everted vesicles enriched in plasma membrane, it has been shown that a metal efflux pump is present in the Leishmania plasma membrane. Like MRPA, this efflux pump also recognizes the metal conjugated to thiols such as GSH and TSH [127] and requires ATP. The identity of this efflux pump is still unknown even 10 years after its discovery. Further, it also appears that this efflux system does not play a significant role in antimony resistance, as the transport kinetics of the vesicles prepared from sensitive and resistant isolates are similar [157].

 Dfferential gene expression study showed that expression of aquaglyceroporins AQP1, responsible for Sb(III) uptake, was downregulated at both the promastigote and the intracellular amastigote stages in antimony-resistant *L. donovani* isolates from Nepal [121]. The mRNA of AQP1 has also been shown to be decreased in antimony-resistant mutants of several Leishmania species.

### 3.5. Changes in the Cytoskeleton

Microtubules are dynamic cytoskeleton polymers consisting of repeating *α*-/*β*-tubulin heterodimers along with *α*-tubulin, and are vital for cell shape, growth and differentiation of Leishmania [158]. Altered expression, polymerisation and cellular distribution of *α*-/*β*-tubulin and apoptosis-like cell death in arsenite resistant Leishmania donovani promastigotes. Expression of *α*-tubulin is similar in both wild-type promastigotes and arsenite-resistant mutants. A twofold increased sensitivity of a mutant resistant to Paclitaxel (known to promote tubulin assembly) is found to decrease the expression of *α*-tubulin in arsenite-resistant mutant promastigotes [159]. On the other hand, the expression level of *β*-tubulin is higher in both stages of an arsenite-resistant mutant than in the wild-type [160], while *α*-tubulin expression is upregulated in the amastigote stage only and is unaltered in the promastigote stage. Although Paclitaxel treatment significantly increases the expression of b-tubulin in resistant promastigotes, it has no effect on c-tubulin expression in either strain, either before or after differentiation [160]. Further, arsenite treatment has been shown to decrease the expression of alpha- and betatubulin in wild-type promastigotes, while expression remains unaltered in an arsenite-resistant mutant [161]. Since tubulin synthesis is regulated by the unpolymerized tubulins, and arsenite has been shown to inhibit microtubule polymerization in the parasite, arsenite may decrease the synthesis of tubulins by inhibiting polymerization. It is noteworthy that phosphorylation of *α*- and *β*-tubulin is highly increased in the arsenite-resistant mutant [162]. Phosphorylation of tubulins could directly affect the dynamics of tubulin assembly and regulate and affect several signal-transduction pathways [163]. Since As and Sb are both are metalloid and mutual cross resistance has been seen in some Leishmania mutants, it could be speculated that tubulin may play an important role in Sb resistance.

### 3.6. Resistance at the Level of Host

The immune status of Leishmania infected patients has long been known to affect drug efficacy. This has proven to be of particular importance in relation to pentavalent antimonial treatment of DCL [164] and coinfections with HIV in the visceral form [165, 166], where there is both an absence of a specific T-cell mediated immune response and mutual exacerbation of infection. The basis for this lack of activity of pentavalent antimonials has been explored in immunodeficient mouse models for which the effects are probably due to deficiencies of both Th1-cell-mediated and macrophage responses [90, 167]. The introduction of highly active antiretroviral therapy [168] again suggesting an important role for CD4 lymphocytes in preventing relapses and controlling the infection.

It was further shown by our group that antimonials activate important signaling pathways of host immune cells like macrophage to induce ROS and NO that ultimately leads killing of intracellular parasites [78]. Interestingly, SAG can also induce the generation of gamma interferon from splenic lymphocytes and the proliferation of splenocytes [169]. Therefore, it was necessary to decipher the role played by the host cell, if any, in Sb unresponsiveness. Further endeavor in this direction by our group revealed that resistant parasites strongly increase expression of host's P-gp and MRP1 transporters on the surface of infected macrophages resulting in Sb clearance from the host cells in the course of *in vitro* as well as *in vivo* experimental infection. Moreover, studies performed on patient samples from Sb-resistant infection areas unequivocally indicate that a similar phenomenon occurs during natural human infection. In contrast to infection with Sb-sensitive *L. donovani* isolates, infection with Sb-resistant *L. donovani* isolates upregulates the multidrug resistance-associated protein 1 (MRP1) and the permeability glycoprotein (P-gp) in host cells, thus inhibiting intracellular drug accumulation [170]. Indeed, it is well established that monocytes do not harbor parasites at the active stage of the disease. In spite of this, peripheral blood monocytes from Sb(V) resistant VL patients upregulate P-gp and MRP1. Therefore, it is likely that soluble and circulating parasite antigens can cause upregulation of expression of these transporters. This is supported by our findings that formalin-fixed Sb resistant *L. donovani* or even extracts from Sb resistant *L. donovani* strains can induce upregulation of MRP1 and P-gp in uninfected murine macrophages and reduce Sb accumulation following SAG treatment. Thus the resistance mechanism may operate in different cells of parasite reservoirs even in the absence of parasite replication *in situ*. Our results also show that inhibitors of P-gp and MRP1 could restore sensitivity toward Sb not only *in vitro* but also *in vivo*. In animal models, inhibition of the proteins MRP1 and P-gp by lovastatin reverses their action on drug accumulation and allows them to escape a fatal outcome. These results indicate that lovastatin, which can inhibit P-gp and MRP1, might be beneficial for reverting Sb resistance in VL. 

 A recent study [171] by our group has shown that antimony sensitive and resistant clinical isolates of *L. donovani* differentially regulate activation of dendritic cells (DCs). SAG-induced signaling pathway associated with DC activation/maturation is selectively targeted by antimony resistant *L. donovani* infection. In contrast to antimony sensitive *L. donovani*, antimony resistant *L. donovani* infection inhibits SAG-induced proinflammatory cytokine secretion as well as upregulation of costimulatory molecule and MHC expression in DCs. Antimony resistant *L. donovani* mediates these inhibitory effects in DCs by blocking SAG-induced activation of the PI3K/AKT and downstream NF-*κ*B pathway. In addition, the suppression of NF-*κ*B activation by antimony resistant *L. donovani* results in inhibition of SAG-induced *γ*GCS heavy-chain (*γ*GCS_hc_) gene expression in DCs. Regulation of host *γ* GCS_hc_ expression and, therefore, of host GSH level by antimony resistant *L. donovani* is important in the view of antimony resistance in LD infection. This study establishes a key role for NF-*κ*B in antimony resistant *L. donovani* -mediated suppression of DCs. Notably, antimony resistant but not antimony sensitive *L. donovani* induces increased IL-10 secretion by DCs. IL-10, a potent suppressor of antileishmanial immunity, is known to minimize responsiveness to SAG. Therefore, increased IL-10 production may play a critical role in disease pathogenesis in the host infected with antimony resistant *L. donovani*. Studies are underway to confirm whether the inhibition of SAG-induced signaling pathways observed in antimony resistant *L. donovani* infected DCs is due to lack of accumulation of the drug itself (as observed previously in case of macrophage system) or due to the effect of antimony resistant *L. donovani* infection.

### 3.7. Antimonials for Cancer

The immune system performs meticulously balanced and harmonious functions and thus protects the host from any undesirable foreign insult. Despite the existence of a multifunctional immunosurveillance process, immunocompetent individuals develop cancer. Cancer induces immense local immunosuppression and global immunosuppression in late stage. Antimonials possess immunomodulatory activity, can activate multiple signaling pathways including NF*κ*B [78], and are also able to modulate intracellular redox balace [39]. Antimonial has been shown to activate T cells, and ameliorate renal cell carcinoma in combination with IL-2 [172]. SAG as well as antimony trioxide have also been shown to possess antileukemic activity [85, 173–175]. Since antimony is cheap and shows both direct action as well as indirect action on both immune cells and tumor cells, therefore antimony compounds are being tried clinically for cancer therapy mainly against leukemia.

 At present novel cost-effective delivery systems for antimonials using liposome and cyclodextrin are being developed by Frezard's group and are showing enhanced efficacy. Interestingly cyclodextrin-based [176] antimony delivery has been found to be orally active. These formulations will not only improve therapeutic use of antimony for leishmaniasis but also for other diseases.

### 3.8. Other Available Drugs 

#### 3.8.1. Amphotericin B

Conventional by, amphotericin B has been used as a second-line treatment for VL since the 1960s. This drug exhibits an excellent antileishmanial activity with >90%–95% cure rates in Indian VL cases. The routine scheme of conventional amhotericin B is 1 mg/kg administered on alternate days for a total of 30 days. However, a recent study in India showed 96% cure rates with a dose of 0.75 mg/kg/day for 15 days [176]. Major disadvantages of conventional amphotericin B are its prolonged administration and the frequent adverse effects, such as infusion-related fever and chills, nephrotoxicity, and hypokalemia, which necessitate administration in hospital [176]. Conventional amphotericin B is used extensively in India for cases unresponsive to antimonials or even as a first line drug. However, outside India this drug does not offer any advantage over pentavalent antimonials. 

 Unresponsiveness and relapses occur rarely, except among HIV-infected patients. In this population, secondary episodes of VL are common and are attributed mainly to relapse but also to reinfection [177]. A recent study failed to disclose decreased susceptibility among *Leishmania* parasites collected from HIV-infected patients during repeated VL episodes (mean follow-up period: 35.6 months; range: 3–137 months), despite repeated courses of amphotericin B.

#### 3.8.2. Miltefosine

Miltefosine (hexadecylphosphocholine) is the first orally administered drug for VL and the latest to enter the market. This agent is associated with high efficacy rates, including cases unresponsive to antimonials [178, 179]. In a phase IV multicenter trial in India of 1132 adults and children with VL treated with miltefosine, cure rates were 82% per intention-to-treat analysis and 95% per protocol analysis [180]. In this study, 3% of patients developed adverse effects, mainly gastrointenstinal toxicity, and elevated hepatic transaminases and creatinine [180]. Data from phase IV clinical trials in India involving domiciliary treatment with miltefosine along with weekly supervision suggested a doubling in the relapse rate against miltefosine [180]. So far, miltefosine is licenced in India, Germany, and Colombia. The scheme of miltefosine treatment is 100 mg/kg/day for 28 days in adults weighing ≥50 kg, 50 mg/kg/day in adults <50 kg, and 2.5 mg/kg/day in children (maximum dose: 100 mg/day). Major concerns for the wide use of miltefosine include its teratogenic potential and its long half-life (approximately 150 hours) which may facilitate the emergence of resistance. Miltefosine is strictly forbidden in women of child-bearing age who may become pregnant up to two months following drug discontinuation. In India miltefosine is available over the counter, a fact that may expose this drug to misuse and emergence of resistance. Once generated, resistant parasites could spread rapidly, endangering the life span of miltefosine in a country where it is needed most [7]. 

The exact antileishmanial mechanism of miltefosine remains largely unknown. The intracellular accumulation of the drug appears to be the critical step for its action. The intracellular accumulation of miltefosine includes the following steps: binding to plasma membrane, internalization in the parasite cell (two proteins, the miltefosine transporter LdMT and its beta subunit LdRos3, are the most significant), and intracellular targeting and metabolism [181]. It has been found that miltefosine induces an apoptosis-like cell death in *L. donovani* by producing numerous defects [181]. Miltefosine also induces several immunologic and inflammatory effects on macrophages. In animal models, miltefosine does not require T-cell-dependent immune mechanisms in order to act, indicating that this agent can be used in T-cell-deficient patients [182]. Recently, it was found that miltefosine enhanced IFN-*γ* receptors and thus IFN-*γ* responsiveness in *L. donovani*-infected macrophages; in the same model, miltefosine induced an IL-12-dependent Th1 response and reversed the Th2 response to Th1 response [183]. 

Resistance to miltefosine may emerge easily during treatment due to single point mutations. Decrease in drug accumulation is the common denominator in all miltefosine resistant Leishmania lines studied to date, and this could be achieved through decreased uptake, increased efflux, faster metabolism, or altered plasma membrane permeability; the first two mechanisms have been already described in models of experimental miltefosine resistance [184]. Two proteins, miltefosine transporter LdMT and its specific beta subunit LdRos3, form part of the miltefosine translocation machinery at the parasite plasma membrane, and are required for miltefosine uptake [181]. Experimental mutations at LdMT or LdRos3 rendered the parasites remarkably less sensitive to miltefosine, and this resistance persisted *in vivo*; cross-resistance with other antileishmanials was not detected [185]. The overexpression of ABC transporters is another mechanism for acquisition of miltefosine resistance, through reduction of the drug intracellular accumulation [185]. Recently, a novel flavonoid derivative was designed and it was shown that the use of suboptimal doses in order to overcome the overexpression of LtrMDR1 (a P-glucoprotein-like transporter belonging to the ATP-binding cassette superfamily) was associated with a fourfold increase of intracellular miltefosine accumulation in the resistant Leishmania lines [186]. Furthermore, modifications in lipid compositions of membranes and sterol biosynthesis have been detected in miltefosine-resistant L. donovani promastigotes [187]. Since membrane fluidity and permeability are influenced by lipid composition, their modification may affect drug-membrane interactions [187]. A case of a healthy patient with VL, who relapsed 10 months after successful treatment with miltefosine for 28 days, was reported recently [188].

#### 3.8.3. Paromomycin

Paromomycin (aminosidine) is an aminoglycoside with antileishmanial activity. In a phase III study of VL in India, this drug was associated with 94.6% cure rates, similar to amphotericin B [189]. Adverse effects were more frequent in the paromomycin-treated group compared with the amphotericin B-treated group (6% versus 2%, resp.); included increased hepatic transaminases, ototoxicity, and pain at injection-site [189]. Currently, paromomycin is under phase IV clinical trials. Paromomycin is inexpensive but requires daily intramuscular injections for 21 days [176]. 

Paromomycin inhibits protein synthesis and modifies membrane fluidity and permeability. An *in vitro* study showed that following a 72-hour exposure of *L. donovani* promastigotes and amastigotes to paromomycin, the mitochondrial potential was decreased, which indicates that mitochondria are the targets of the drug [190]. In laboratory-derived resistant parasites developed through serial-passage increasing-drug concentrations, paramomycin uptake was decreased compared to the wild-type parasite, in association with inhibition of protein synthesis; no cross-resistance with other antimonial agents was detected [190]. Since paromomycin is an aminoglycoside, it is possible that resistance will emerge rapidly if used as monotherapy.

#### 3.8.4. Combination Regimens

The rational for using combination regimens with different resistance mechanisms over monotherapy relies on the expected enhanced efficacy (through synergy or additive activity without drug interaction), shorter treatment duration, less toxicity, improved compliance, reduced likelihood of emergence of resistance, and reduced costs. A combination policy for VL is supported by the fact that antileishmanial drugs belong to different chemical classes [195]. Recent studies have investigated this option. In a retrospective study conducted among Sudanese patients with VL, it was found that combination of sodium stibogluconate and paromomycin administered for 17 days was associated with higher cure and survival rates compared to sodium stibogluconate monotherapy administered for 30 days (44%–86% lower odds of death in the combination group) [191]. Combinations of miltefosine with amphotericin B, paromomycin or pentavalent antimonials have been evaluated in an *in vivo* model and this revealed that the combinations of miltefosine with amphotericin B or paromomycin were efficacious [192]. These preliminary data justified a recent study in Bihar, India, comparing 5 mg/kg of liposomal amphotericin B administered once (group A; 45 patients), 5 mg/kg of liposomal amphotericin B administered once plus miltefosine for either 10 days (group B; 46 patients) or 14 days (group C; 45 patients), 3.75 mg/kg of liposomal amphotericin B administered once plus miltefosine for 14 days (group D; 45 patients), and 5 mg/kg of liposomal amphotericin B administered once followed by miltefosine for 7 days (group E; 45 patients); in this study, similar final cure rates (91%–98%) were noted in all treatment groups. These data indicate that a single dose of liposomal amphotericin B followed by 7–14 days of miltefosine is active against Indian VL [193]. In this study, all patients were treated in an outpatient setting. Large, randomized-controlled trials are required before adaptation of combination regimens. 

Several combination regimens with investigational agents have been tested *in vitro* and in animal models [194]. The plant-derived immunostimulant agent picroliv has no antileishmanial activity; however, when administered with half-dose miltefosine, it increases significantly the activity of the latter [195].

### 3.9. Peroxovanadium Compounds towards the Reversal of Antimony Resistance

There are reports that peroxo- and diperoxo-vanadate compounds are potential antileishmanial agents in a number of *in vitro* and *in vivo *assays [196, 197]. The peroxide of vanadium (PV, a mixture of vanadate and H_2_O_2_) is an insulinomimetic agent and a potent inhibitor of protein tyrosine phosphatase (PTP) [198–202]. Inhibition of PTP by peroxovanadate can modulate the leishmanicidal response by inducing microbicidal effector molecules (like NO, ROS) along with IFN-*γ* [196, 197]. The peroxovanadate compounds that are used against experimental infection contain 1,10-phenanthroline, pyridine-2-carboxyl or bipyridine as ancillary ligands [197, 198, 202]. A number of chemically defined PV derivatives, each containing an oxo ligand, one or two peroxo anions in the inner coordination sphere of vanadium, and an ancillary ligand, are equally potent PTP inhibitors stable in aqueous solution [202]. These can activate the insulin receptor kinase, mimic insulin biological action *in vivo *[198], and also activate the response of immune cells [203]. Both in human and mice, the severity of visceral leishmaniasis have been most closely associated with increased levels IL-10, where the ratio of IFN-*γ* : IL-10 is the important denominator for the protection [204–206]. Thus peroxovanadate complexes appear to possess the potential to become antileishmanial agents. 

**Figure 4:**
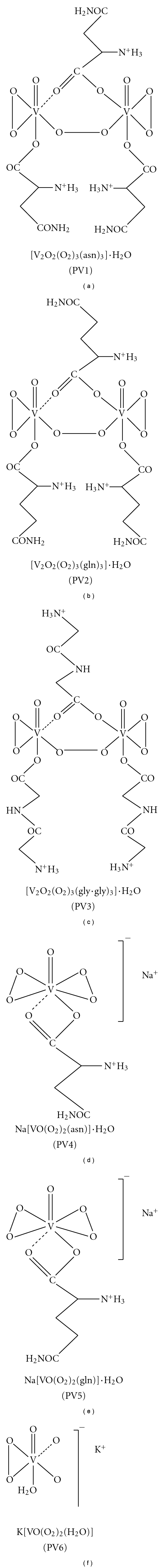
Structures and formulae of the PV compounds [218].

We tested a number of vanadium compounds, which are different from those used against experimental infection, with respect to their ancillary ligands in the coordination sphere of the compounds (Figure 4), to get the potent variety that may be of therapeutic application against leishmaniasis. Another compelling reason to test vanadium compounds is that vanadate is an inhibitor of P-gp [207–209], which is well related to Sb-resistace in leishmaniasis [210]. We have studied six peroxovanadate compounds ((three dinuclear triperoxovanadate (TPV, (a)–(C) in Figure 4) complexes and three mononuclear diperoxovanadate (DPV, (d)–(f) in Figure 4) complexes). Our study showed that one of the mononuclear diperoxovanadate compounds (designated as PV6) is highly effective in killing intracellular *Leishmania* parasites. When PV6 was injected together with SAG, the combination showed enhanced antileishmanial activity *in vivo *in terms of reduction in organ parasite burden in BALB/c mice infected either with SAG sensitive or SAG unresponsive strain. Our study also showed that immune parameters like antileishmanial T cell response as also ROS and NO production were enhanced in response to the combination treatment. Most importantly, such therapy allowed increased IFN-*γ* production with concomitant decrease in IL-10 generation, an indicator for favorable antileishmanial immune. 

## 4. Strategies Available to Combat Drug Resistance

### 4.1. Drug Resistance Monitoring

Improved methods to monitor drug resistance are essential that determine either the (i) phenotypic sensitivity of parasite isolates or (ii) molecular changes that indicate alterations in either the drug target or mechanisms that alter the intraparasite level of active drug. There are problems with both approaches. First, the determination of drug sensitivity of clinical isolates is open to the criticism that pathogen adaptation from host to culture media immediately selects for a subpopulation of pathogens best suited for growth in that medium. The drug sensitivity of parasites must therefore be tested as soon as possible after isolation from the patient using defined agreed protocols. Although promastigote assays are easiest and quickest, this assay is not predictive for pentavalent antimonials, and possibly not for other antileishmanials also, for example, paromomycin, pentamidine, and miltefosine. The amastigote-macrophage assay is currently the only model able to correlate clinical response to the sensitivity of the isolate, as demonstrated in relation to pentavalent antimonials [3]. Axenic amastigotes are sensitive to antimonials but adaptation of isolates is both too selective and too lengthy a process to be used in this type of assay [211]. Second, the ability to develop molecular probes or PCR-based diagnostics to monitor the development and spread of drug resistance is severely limited by a lack of knowledge of the molecular and biochemical mechanisms of action and resistance of most antileishmanial drugs, especially in clinical isolates [114].

### 4.2. Monitoring Therapy

The introduction of an oral drug for leishmaniasis offers advantages of improved compliance, self administration, and reduced costs. In the phase IV trial for miltefosine, a 7-day supply is issued to patients who have to return to the clinic each week for examination and resupply. For drugs like miltefosine which have a long half-life and a propensity for selection of resistant forms, the monitoring of daily dosing and the completion of a course of treatment are essential. The directly observed treatment strategy for tuberculosis chemotherapy has been successfully introduced in India by the Revised National TB Control Programme in 1997 (http://www.who.int/gtb/publications/globerep/index.html). The potential for use of a parallel system for the control of leishmaniasis, for miltefosine at present possibly also for sitamaquine in future, should be considered [114].

### 4.3. Cost and Distribution of Drugs

The approximate cost of treatment of a patient with VL in India is given in Progressive failure of antimonial drug treatment, which is the only available drug treatment in the public health program in India, has driven most of the VL patients in India towards the private sector. The drugs, including antimonials, amphotericin B, and now miltefosine, can be bought over the counter without restriction on quantity. The cash-starved population buys antileishmanial drugs in instalments, and most do not complete treatment [102] as disease symptoms are alleviated quickly. Considering the cost of drugs, antimonials have been the only drugs that are barely affordable. Miltefosine, which is being used extensively in the private sector, is ~6 times more expensive and it is not mandatory to buy the full course. This is likely to result in widespread underdosing, sharing of doses among patients, and ultimately emergence of resistance to this important and only oral antileishmanial compound. Considering the inability of the majority of the population to purchase and complete a full course of the drug, and the chaotic system of drug marketing, it has been suggested that, miltefosine should be withdrawn from the private sector and made available free through public and/or private health care providers to prolong the effective life of this important drug [7].

### 4.4. Diagnostic Methods

The improvement in noninvasive serological diagnostic methods with high sensitivity and specificity, for example, DAT, K39, and Katex (urine dipstick), is a major advance in the control of leishmaniasis [212, 213]. In the context of chemotherapy what is required is a noninvasive diagnostic kit that can be used to monitor drug response and determine cure in patients. Antibody levels do not always indicate active infection, vary between individuals, and are of no use in HIV/VL coinfection cases. Antigen detection is far more important for monitoring drug response; further improvement of methods such as the Katex kit [214] might be of particular interest in this case. The variation in species sensitivity has greatest clinical significance in Central and South America, where the distribution of *L. mexicana*, *L. amazonensis*, *L. panamensis*, *L. braziliensis*, and other members of these groups overlap. The distinctive amastigote and macrophage interaction of mexicana group parasites makes some level of diagnosis by microscopy feasible for trained staff. Molecular tools that have been developed need to be implemented to distinguish the braziliensis group species.

### 4.5. Combination Therapies

Drug combinations have proven to be essential features of antimicrobial treatment through design or use to (i) increase activity through use of compounds with synergistic or additive activity, (ii) prevent the emergence of drug resistance, (iii) lower required doses, reducing chances of toxic side effects and cost, or (iv) increase the spectrum of activity, for example, the use of an antileishmanial with either an antiinflammatory or immunodulator in cutaneous leishmaniasis. Previous studies on drug combinations for VL, for example allopurinol plus sodium stibogluconate and paromomycin plus sodium stibogluconate [215]. The use of combinations to combat resistance has been well rehearsed in antimalarials; studies to identify such combinations are new for leishmaniasis; limited studies are under way to examine interactions of miltefosine with other antileishmanials to identify suitable combinations. Bryceson [216] advocated the examination of combinations of strong antileishmanials with “weak” drugs (e.g., azoles); this is an approach also used in malaria treatment, for example, the inclusion of clindamycin or azithromycin in combinations. A combination therapy also needs to be evaluated for safety and optimized for either concomitant or sequential administration of component drugs.

### 4.6. Resistance Reversal Agents

The strategy to reverse resistance has long been discussed in relation to chloroquine resistance in *Plasmodium falciparum *and produced interesting experimental results without any clinical impact. In laboratory studies on *Leishmania, *a series of sesquiterpenes have been shown to reverse drug resistance due to P-glycoproteins in an *L. tropica *clone. Another study suggested a strategy of inhibition of thiol levels by coadministration of antimony with an inhibitor of glutathione biosynthesis.

### 4.7. New Targets, New Drugs

There are few better ways to avoid drug resistance than to have an adequate armory of drugs with different targets and no cross-resistance. Although miltefosine has been approved for use in the treatment of VL in India, paromomycin is moving through phase III trials in India and Africa, and sitamaquine remains in phase II development for leishmaniasis [217], all these drugs have clear limitations of toxicity, long courses of treatment, or parenteral administration. More clearly defined criteria of the needs and target profiles for new drugs and new treatments are required.

## 5. Conclusions

The control of VL globally is challenged by the widespread emergence of antimonial resistance in India. In the last decade new formulations of conventional antileishmanial drugs as well as new agents became available. The wide use of the oral agent miltefosine was hampered by the potential for teratogenicity and emergence of resistance. Combination regimens should be evaluated in large trials. During last few years several mechanisms of in-field antileishmanial resistance were identified. Understanding their molecular and biochemical characteristics will lead to the design of new drugs and also the molecular surveillance of resistance. In order not to jeopardize the life span of available antileishmanials, their delivery, clinical response, and resistance should be monitored. Overall the development of antileishmanials has been generally slow; new drugs are needed.

## Figures and Tables

**Table 1:** Changing therapeutic response to pentavalent antimonials (Adapted from T. K. Jha, 2006 [219]).

Study	Dose (mg/kg/day)	Duration (days)	No. of courses	No. of cases	Unresponsiveness (%)
Jha, (1980) [220]	10	10	1	200	17

Thakur et al., (1984) [221]	20	20	1	64	8
		>20	1	62	0

Jha, (1986) [222]	Child-20	Fresh-30	1	Fresh-73	1.1
	Adult-10	Relapse-60	1	Relapse-17	
		Slow	1		
		response-42			

Thakur et al., (1988) [223]	10	40	1	371	26
	15		1		14
	20		1		3

Jha, (1992) [224]	20	30	1	252	27.1

Jha, (1995) [225]	20	30	1	32	25

Jha, (1998) [226]	20	30	1	30	37

Thakur et al., (1998) [227]	20	30	1	80	54

Sundar et al., (2001) [102]	20	30	1	184	60
